# Patterns of richness, diversity and abundance of an odonate assemblage from a tropical dry forest in the Santiago Dominguillo Region, Oaxaca, México (Insecta: Odonata)

**DOI:** 10.3897/BDJ.9.e60980

**Published:** 2021-04-22

**Authors:** Enrique González-Soriano, Felipe A. Noguera, Cisteil X. Pérez-Hernández, Santiago Zaragoza-Caballero, Leonardo González-Valencia

**Affiliations:** 1 Departamento de Zoología, Instituto de Biología, UNAM, Ciudad de México, Mexico Departamento de Zoología, Instituto de Biología, UNAM Ciudad de México Mexico; 2 Estación de Biología Chamela, Instituto de Biología, UNAM, San Patricio, Jalisco, Mexico Estación de Biología Chamela, Instituto de Biología, UNAM San Patricio, Jalisco Mexico; 3 Instituto de Investigaciones en Ecosistemas y Sustentabilidad, UNAM, Morelia, Michoacán, Mexico Instituto de Investigaciones en Ecosistemas y Sustentabilidad, UNAM Morelia, Michoacán Mexico

**Keywords:** assemblage structure, phenology, taxonomic distinctness

## Abstract

A study on the patterns of richness, diversity and abundance of the Odonata from Santiago Dominguillo, Oaxaca is presented here. A total of 1601 specimens from six families, 26 genera and 50 species were obtained through monthly samplings of five days each. Libellulidae was the most diverse family (21 species), followed by Coenagrionidae (19), Gomphidae (4) and Calopterygidae (3). The Lestidae, Platystictidae and Aeshnidae families were the less diverse, with only one species each. *Argia* was the most speciose genus with 11 species, followed by *Enallagma*, *Hetaerina*, *Erythrodiplax* and *Macrothemis* with three species each and *Phyllogomphoides*, *Brechmorhoga*, *Dythemis*, *Erythemis* and *Orthemis* with two species each. The remaining 17 genera had one species each. *Argia pipila* Calvert, 1907 and *Leptobasis vacillans* Hagen in Selys, 1877 were recorded for the first time for the state of Oaxaca. We also analysed the temporal patterns of taxonomic and phylogenetic divergence for the Santiago Dominguillo Odonata assemblage: the Shannon diversity value throughout the year was 21.07 effective species, while the Simpson diversity was 13.17. In general, the monthly phylogenetic divergence was higher than expected for taxonomic distinctness, and lesser for average taxonomic distinctness. Monthly diversity, evenness and taxonomic divergence showed significant positive correlations (from moderate to strong) with monthly precipitation values. The analysis of our results, however, indicates that an increase in rainfall not only influences the temporal diversity of species, but also the identity of supraspecific taxa that constitute those temporal assemblages, i.e. there is an increase in temporal phylogenetic divergence.

## Introduction

Tropical forests are the most important reservoirs of terrestrial biodiversity around the world. Despite there being many studies in these ecosystems,most of them focus on tropical wet forests (TWF), with less attention to the tropical dry forests (Mooney et al. 1995). Tropical dry forests (abbreviated herein as TDF) are seasonal ecosystems in which precipitation occurs only during a portion of the year and the decrease in rainfall causes changes in the patterns of greenish/senescence of the forest, resulting in a reduction of productivity and availability of resources for animals and, consequently, in the biological activity in the forest during the dry season (Martínez-Hernández et al. 2019). TDF are the tropical community with the greatest extension in Mexico, stretching along the Pacific coast from Sonora south to Chiapas in the Mexico-Guatemala border, with some intrusions in Central Mexico, northern Veracruz and the Yucatan Peninsula (Búrquez and Martnez-Yrízar 2010, Trejo 2010). The relevance of the TDF has been recognised by many authors as very rich ecosystems with an abundance of endemic animal and plant species, which are, however, at great risk due to deforestation and human activities (e.g. cattle ranching and agriculture, particularly in Mesoamerica, (Banda 2016, Miles et al. 2006, Sánchez-Azofeifa et al. 2005). Therefore, documenting the biodiversity of the TDF is an urgent task for the proper management and conservation of this ecosystem.

Seasonal fluctuations of richness and abundance of insects inhabiting tropical forests has been documented elsewhere (e.g. Kannagi et al. 2016, Kishimoto-Yamada and Itioka 2015, Wolda 1988). In Mexico, it has been monitored in some detail at localities such as the Chamela-Cuixmala Biosphere Reserve in Jalisco State (García-Aldrete and Ayala-Barajas 2004), but such studies are scarce for other sites. With the aim of filling this gap, in 1997, a group of entomologists from the Institute of Biology of the National Autonomous University of Mexico (UNAM) started a long-term project to document the richness and distributional patterns of selected groups of insects associated with the TDF in Mexico. Most of the localities studied are distributed along the Pacific slope from Sonora to Oaxaca, with a few studies in south-central Mexico (e.g. González-Soriano et al. 2008, González-Soriano et al. 2009, Noguera et al. 2002, Noguera et al. 2007, Noguera et al. 2009, Noguera-Martínez et al. 2012, [Bibr B6283078][Bibr B6283817]).

Odonata was included in that project as a “test group”, with the aim of comparing if their local assemblages vary in a similar way as those exhibited by other insect groups that are more directly associated with plants (e.g. Cerambycidae, Lampyridae, Syrphidae and Apidae). Dragonflies are predaceous insects that do not depend directly on plants from a trophic point of view, but forests can provide certain requirements for the adults, such as optimal microclimates for effective thermoregulation, conditions for an optimal foraging and a provision for nocturnal roosting or daytime shelter from both inclement weather and predators (Corbet 1999, Corbet et al. 2006, Paulson 2006). Vegetation can also be used as mating areas and feeding perches (Buchwald 1992).

To date, only a few studies documenting the patterns of diversity and abundance of Odonata have been done in the Mexican TDF: one at the Chamela Biosphere Reserve in Jalisco (González-Soriano et al. 2004) and another two at the Sierra de Huautla in Morelos and the Sierra de San Javier in Sonora (González-Soriano et al. 2009, González-Soriano et al. 2008). Additionally, Novelo-Gutiérrez & Gómez-Anaya (Novelo-Gutiérrez and Gómez-Anaya 2008) included a couple of sites with TDF (Pinolapa and Aguililla) in their study on the altitudinal distribution of Odonata assemblages in the Sierra de Coalcoman in Michoacan.

We are presenting here the results of a study on the patterns of richness, diversity and abundance of an Odonata assemblage in Santiago Dominguillo, Oaxaca, Mexico, which was originally carried out between November 1997 and October 1998.

## Material and methods

The study area was the vicinity of the town of Santiago Dominguillo (referred to herein as Dominguillo), in the north-eastern part of the State of Oaxaca (17.6484, -96.91117, 760 m a.s.l.), south of the Tehuacan-Cuicatlan Biosphere Reserve. The area belongs to what is known as the Floristic Province of the Tehuacan-Cuicatlan Valley, which is considered part of the xerophytic Mexican region (Rzedowski 1991).

The climate is semi-warm type according to the Köppen climate classification modified by García (García 1988). Average annual precipitation is 521.5 mm and air temperature is 25.2°C (Jaramillo-Luque and González-Medrano 1983). The dominant vegetation in the area is TDF and *Lysiloma divaricatum* (Jacq.), *Bursera aptera* Ramírez, *B. morelensis* Ramírez, *B. schlechtendalii* Engl, *Cyrtocarpa procera* Kunth, *Pachycereus weberi* (J.M. Coult.) Backeb, *Escontria chiotilla* (F.A.C.Weber ex K.Schum.) Rose and *Ceiba parvifolia* Rose (Jaramillo-Luque and González-Medrano 1983, Ochoa 2001) are the dominant trees. Gallery forest is also present along rivers, with trees as tall as those found in contiguous TDF. Some of the flat areas in the region have been converted into pasture lands and mango plantations.

The area belongs to the hydrological region No. 28, which corresponds to the Papaloapan basin, a river draining into the Gulf of Mexico and the site corresponds to the Rio Grande sub-basin, which enters the Tehuacan-Cuicatlan Reserve from the south.

**Sampling methods and regimes.** Most samplings were done around the Rio Las Vueltas (also known as Rio de las Vueltas) and other minor tributaries around the Cuicatlan-Dominguillo vicinity, including the towns of Santiago Dominguillo (N 17.6484, W -96.91117) and San Pedrito Chicozapote (N 17.77218, W -96.9445), with an occasional collection also in Rio Grande, Presa Derivadora Matambo (N 17.6484, W -96.9117). Fieldwork was carried out monthly from November 1997 to October 1998. Samplings were done for five days every month, from 09:00 to 15:00 h (10:00 to 16:00 h during daylight savings time).

*Diversity analysis*. We analysed the diversity of the Odonata assemblage of Santiago Dominguillo and its temporal pattern. The assemblage structure was analysed through the abundance (number of individuals), richness (number of species observed, ^0^D), Shannon diversity (exponential of Shannon Index, ^1^D) and Simpson diversity (inverse of Simpson Index, ^2^D) ([Bibr B6275267][Bibr B6283000]). The unit of measurement for ^0^D and ^1^D is the *number of effective species* or Hill numbers: ^1^D indicates the effective number of species equally abundant within an assemblage or community, while ^2^D is the effective number of the most abundant or dominant species equally abundant. These metrics are mere modifications of the indices used in previous works to analyse diversity (e.g. González-Soriano et al. 2008) and we used them in order to obtain direct measures of diversity that can be interpreted from a biological perspective (number of effective species instead of *bits* or *nats*) (Cultid-Medina and Escobar 2019).

The maximum expected richness value of diversity was estimated by non-parametric abundance, based Chao 1-bias corrected estimator (Chao 2005), as well as the expected diversity for ^1^D and ^2^D using the estimators proposed by Magurran (Magurran 1988) and Chao et al. (Chao et al. 2013). All these calculations were done using the Spade R package (Chao et al. 2015). We also used the iNext package (Hsieh et al. 2016) to obtain the cumulative species curve for the whole Odonata assemblage from the Dominguillo Region, through the interpolation-extrapolation method proposed by Chao et al. (Chao et al. 2014).

To evaluate temporal diversity patterns for the Dominguillo Odonata assemblage, we performed the same diversity metrics described above (abundance, ^0^D, ^1^D and ^2^D), based on the monthly information occurrence of collected odonates. Additionally, we evaluated the monthly phylogenetic divergence using the taxonomic distinctness (Δ*) and average taxonomic distinctness (Δ+) indices based on the abundance and incidence of the species, respectively (Clarke and Warwick 1998, Clarke and Warwick 1999, [Bibr B6282821][Bibr B6283356]), which were calculated using the *taxondive* and *taxa2dist* functions of the Vegan R package (Oksanen et al. 2015). These measurements indicate that the higher value of Δ* and/or Δ+, the greater phylogenetic distance amongst individuals (Δ*) and species (Δ+) within an assemblage.

The taxonomic distinct is a measure to evaluate the phylogenetic divergence within the communities or assemblages according to the topology of their taxonomic hierarchy and it analyses the pattern of the phylogenetic relationships amongst taxa obtained from a sample or a complete assemblage, i.e. how closely related the species are or how evenly distributed are their evolutionary paths through the taxonomic hierarchy (Clarke and Warwick 1998, Clarke and Warwick 2001). Here, we included the Odonata taxonomic hierarchy: order, superfamily, subfamily, tribe, genus and species. The supraspecific taxonomic arrangement was based on Carle et al. 2015, Davies and Tobin 1985, Dijkstra et al. 2014.

To evaluate if the Dominguillo odonate diversity is affected by or related to abiotic factors, Pearson’s correlation analyses were done between the monthly species diversity (abundance, ^0^D, ^1^D and ^2^D), the monthly phylogenetic diversity (Δ*, Δ+) and the average rainfall and temperature recorded in Dominguillo during the sampling time. All the analyses were done using Past software (Hammer et al. 2001). We also generated a heatmap with *gplots* v. 3.0.4 package of R (Warnes et al. 2012) in order to display the presence and abundance that each species exhibited monthly.

Monthly diversity and phylogenetic divergence analyses allowed us to evaluate how the species diversity and the taxonomic assemblage structure were related to monthly changes of temperature and humidity. In other words, those analyses allowed us to evaluate how the abiotic factors can be associated with the temporal structure of the Odonata community of the TDF.

For the phenological analyses, we considered the period from June-July to September as the rainy season and October to May-June as the dry season. This categorisation was based in the occurrence of individual events of rainfall higher than 15 mm, since events with lower rainfall are intercepted by the canopy (Cervantes 1988). Rainfall values correspond to the climatological norms of the Mexican National Meteorological Service and were obtained from the nearest climatological station of Dominguillo, Oaxaca (CONAGUA (Comisión Nacional del Agua) 2020).

All the material collected was deposited at the CNIN (Colección Nacional de Insectos del Instituto de Biología), UNAM, Mexico City.

## Results

**Figure 1.**
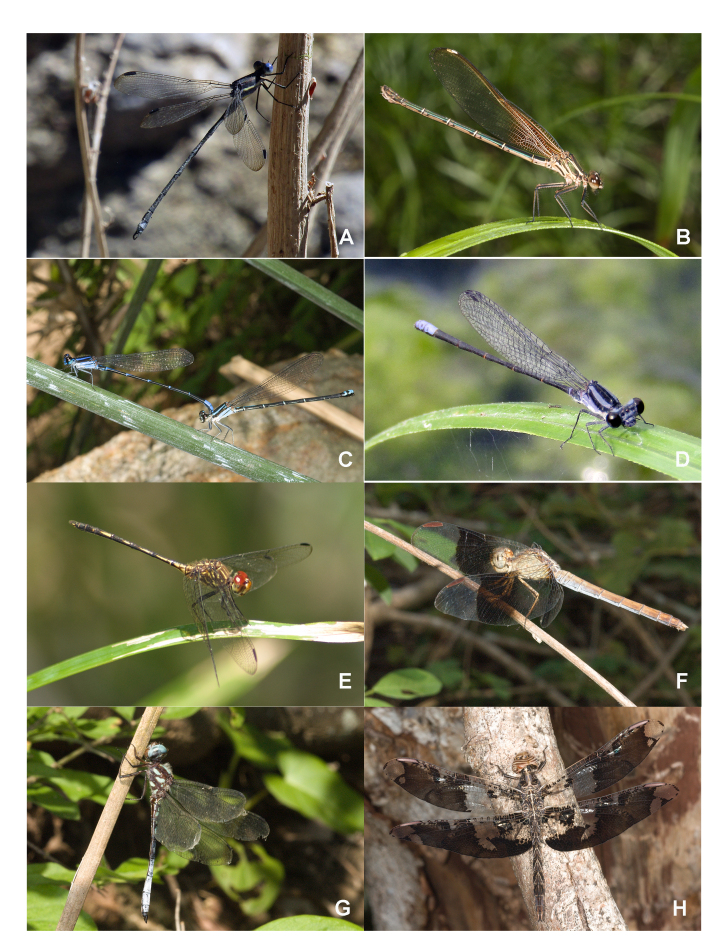
Odonata from Dominguillo, Oaxaca, some examples: **A.** *Archilestes grandis* male (Lestidae); **B.** *Hetaerina americana* female (Calopterygidae); **C.** *Argia oculata* in tandem (Coenagrionidae); **D.** *Argia pulla* male (Coenagrionidae); **E.** *Dythemis sterilis* male (Libellulidae), **F.** *Erythrodiplax umbrata* female (Libellulidae), **G.** *Macrothemis pseudimitans* male (Libellulidae); **H.** *Pseudoleon superbus* male (Libellulidae). Pictures of Enrique González (A, D) and Enrique Ramírez (B, C, E, F, G, H).

List of Odonata species registered from Santiago Dominguillo, Oaxaca, Mexico, including phenology data and number of individuals collected (in parenthesis). Additional information in Suppl. materials 1, 2 and photographs of some species in Fig. 1.

**Lestidae**

*Archilestes grandis* (Rambur, 1842). Jun (1). (Fig. 1)

**Platystictidae**

*Palaemnema domina* Calvert, 1903. Jun (13), Jul (2), Aug (6).

**Calopterygidae**

*Hetaerina americana* (Fabricius, 1798). Nov (41), Jan (23), Mar (30), Apr (11), May (23), Jun (6), Jul (7), Aug (3), Sep (24), Oct (4). (Fig. 1).

*Hetaerina occisa* Hagen *in* Selys, 1853. May (1).

*Hetaerina cruentata* (Rambur, 1842). Jan (2), Mar (11), Apr (7), May (1), Jun (2), Jul (6), Aug (7), Sep (7), Oct (5).

**Coenagrionidae**

*Apanisagrion lais* (Brauer in Selys, 1876). Jul (1).

*Acanthagrion quadratum* Selys, 1876. Jan (1), Apr (4), May (9), Jun (6), Jul (2), Sep (2).

*Argia anceps* Garrison, 1996. Nov (8), Jan (9), Mar (14), Apr (7), May (17), Jun (4), Jul (7), Aug (8), Sep (19), Oct (6).

*Argia extranea* (Hagen, 1861). Nov (2), Jan (2), Mar (10), Apr (2), May (6), Jun (4), Jul (4), Aug (3), Sep (21), Oct (4).

*Argia funcki* (Selys, 1854). May (1), Jun (1), Jul (2), Aug (1).

*Argia harknessi* Calvert, 1899. Jan (1), Mar (5), Apr (1), May (1), Jun (3), Jul (2), Aug (2), Sep (4).

*Argia immunda* (Hagen, 1861). Nov (31), Jan (6), Feb (3), Mar (19), Apr (7), May (7), Jun (2), Jul (4), Aug (2), Sep (2), Oct (4).

*Argia oculata* Hagen in Selys, 1865. Jan (3), Mar (1), Apr (8), May (7), Jun (1), Jul (5), Aug (2), Sep (14), Oct (3). (Fig. 1).

*Argia oenea* Hagen in Selys, 1865. Nov (21), Jan (6), Feb (3), Mar (14), Apr (25), May (28), Jun (6), Jul (4), Aug (4), Sep (7), Oct (6).

*Argia pallens* Calvert, 1902. Nov (6), Jan (8), Mar (8), May (2), Jun (2), Jul (1), Aug (1), Oct (4).

*Argia pipila* Calvert, 1907. Aug (1).

*Argia pulla* Hagen in Selys, 1865. Nov (42), Jan (21), Feb (5), Mar (45), Apr (54), May (85), Jun (19), Jul (9), Aug (6), Sep (20), Oct (5). (Fig. 1).

*Argia tezpi* Calvert, 1902. Nov (6), Jan (8), Feb (7), Mar (29), Apr (15), May (28), Jun (9), Jul (1), Aug (2), Sep (5), Oct (2).

*Enallagma novaehispaniae* Calvert, 1907. Nov (2), Jan (1), Mar (4), Jun (5).

*Enallagma praevarum* (Hagen, 1861). Nov (6), Jan (11), Feb (2), Mar (5).

*Enallagma semicirculare* Selys, 1876. Mar (1).

*Ischnura denticollis* (Burmeister, 1839). Jan (1).

*Leptobasis vacillans* Hagen in Selys, 1877. Apr (1)

*Telebasis salva* Hagen in Selys, 1877. Nov (4), Jan (4), Feb (1), Mar (6), Apr (3), May (3), Jun (4), Aug (7).

**Aeshnidae**

*Anax walsinghami* McLachlan, 1883. Nov (1).

**Gomphidae**

*Erpetogomphus elaps* Selys, 1858. Nov (4), Jun (4), Jul (8), Aug (6), Sep (8), Oct (7).

*Phyllogomphoides danieli* González & Novelo, 1990. Jun (3), Jul (9), Aug (2).

*Phyllogompoides suasus* (Selys, 1859). Aug (3), Oct (2).

*Progomphus clendoni* Calvert, 1905. Jun (1), Jul (4).

**Libellulidae**

*Brechmorhoga mendax* (Hagen, 1861). Nov (1).

*Brechmorhoga praecox* (Hagen,1861). Nov (2), Mar (2), May (5), Jun (8), Jul (9), Aug (7), Sep (4), Oct (1).

*Dythemis nigrescens* Calvert, 1899. Mar (1), Jun (4), Jul (4), Aug (2).

*Dythemis sterilis* Hagen, 1861. Nov (7), Jan (5), Mar (8), Apr (2), May (6), Jun (15), Jul (7), Aug (1), Sep (2), Oct (1). (Fig.1e)

*Erythemis plebeja* (Burmeister, 1839). Jun (1).

*Erythemis vesiculosa* (Fabricius, 1773). May (2), Jun (1).

*Erythrodiplax funerea* (Hagen, 1861). Apr (1), May (2), Jun (2).

*Erythrodiplax fusca* (Rambur, 1842). Nov (12), Jan (4), Feb (3), Mar (8), Apr (4), May (5), Jun (8), Jul (2), Aug (6).

*Erythrodiplax umbrata* (Linnaeus, 1758). Apr (1). (Fig. 1)

*Libellula croceipennis* Selys, 1868. Nov (1), Apr (1), Jun (4), Jul (7), Aug (5), Sep (1), Oct (3).

*Macrothemis hemichlora* (Burmeister, 1839). Nov (1), Apr (2), May (5), Jun (7), Aug (3), Sep (1).

*Macrothemis inacuta* Calvert, 1898. Jun (6), Jul (3).

*Macrothemis pseudimitans* Calvert, 1898. Nov (4), Jan (5), Feb (1), Mar (6), May (1), Jun (9), Jul (6), Aug (3), Oct (3). (Fig. 1)

*Miathyria marcella* (Selys in Sagra, 1857). Jun (3).

*Orthemis discolor* (Burmeister, 1839). Jan (1), Mar (2), May (2), Jul (1), Sep (4), Oct (2).

*Orthemis ferruginea* (Fabricius, 1775). Nov (5), Jan (2), Apr (1), Jun (1), Oct (3).

*Paltothemis lineatipes* Karsch, 1890. Nov (1), May (1), Jul (3), Aug (1), Oct (1).

*Pantala flavescens* (Fabricius, 1798). Nov (1), Aug (1), Oct (1).

*Perithemis mooma* Kirby, 1889. Mar (1), Jul (3), Aug (2).

*Pseudoleon superbus* (Hagen, 1861). Nov (1), Mar (1), May (1), Jun (2), Jul (2), Aug (3), Oct (1). (Fig. 1)

*Tramea onusta* Hagen, 1861. Nov (2).

### Analysis

#### Species richness, abundance and diversity

A total of 1601 specimens from six families, 26 genera and 50 species were collected. Those values represent 50% of the families, 44% of the genera and 31% of the total species previously recorded for the State of Oaxaca (González-Soriano and Novelo-Gutiérrez 2014). Libellulidae and Coenagrionidae were the families with the highest number of species, 21 and 17, respectively, followed by Gomphidae with four, Calopterygidae with three and Lestidae, Platystictidae and Aeshnidae with only one species each (see Table 1). Regarding the number of genera, Libellulidae had the highest number with 13, followed by Coenagrionidae with seven, Gomphidae with three and Lestidae, Calopterygidae, Platystictidae and Aeshnidae with only one genus each. The most speciose genus was *Argia*, with 11 species, followed by *Enallagma*, *Hetaerina*, *Erythrodiplax* and *Macrothemis* with three species each, *Phyllogomphoides*, *Brechmorhoga*, *Dythemis*, *Erythemis* and *Orthemis* with two species each and the rest of the 17 genera with only one species each. *Argia pipila* Calvert, 1907 and *Leptobasis vacillans* Hagen in Selys, 1877 were recorded for the first time in the State of Oaxaca (González-Soriano and Novelo-Gutiérrez 2007, González-Soriano, unpublished data).

**Figure 2.**
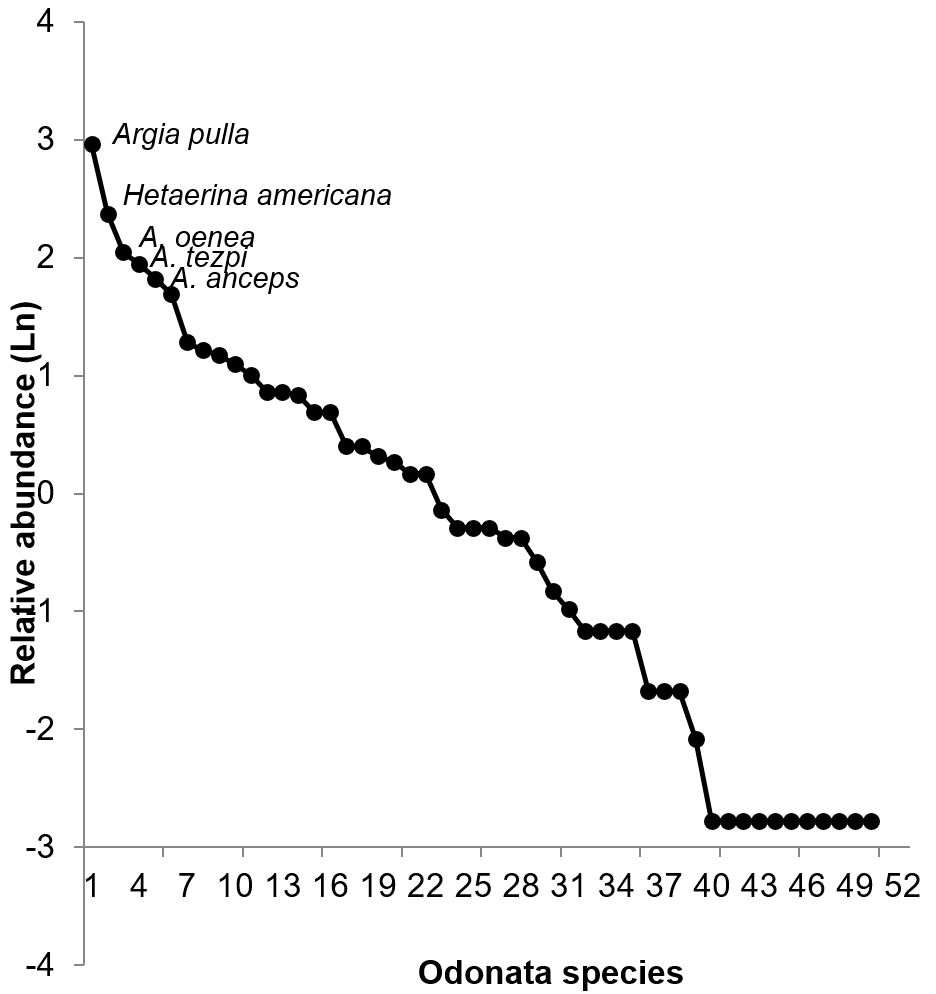
Rank-abundance distribution curve of Odonata species recorded in Santiago Dominguillo, Oaxaca, during 1997-1998.

Species abundance was very heterogeneous. A few species were very abundant, while most were represented by one or few individuals (see Fig. 2). *Argia pulla* Hagen in Selys, 1865 was the most abundant species (311 individuals), followed by *Hetaerina americana* Fabricius, 1798 (172), *Argia oenea* Hagen in Selys, 1865 (124), *Argia tezpi* Calvert, 1902 (112) and *Argia anceps* Garrison, 1996 (99). Those five species represented 51% of the total abundance of the assemblage (818 individuals) and *Argia*, in particular, contributed the highest number of individuals (646). No anisopteran had more than 100 individuals, with *Dythemis sterilis* Hagen, 1861 being the most abundant, with 54 individuals. In contrast, 11 species were represented by only one individual, contributing only 0.68% of the total abundance (Fig. 2).

**Figure 3.**
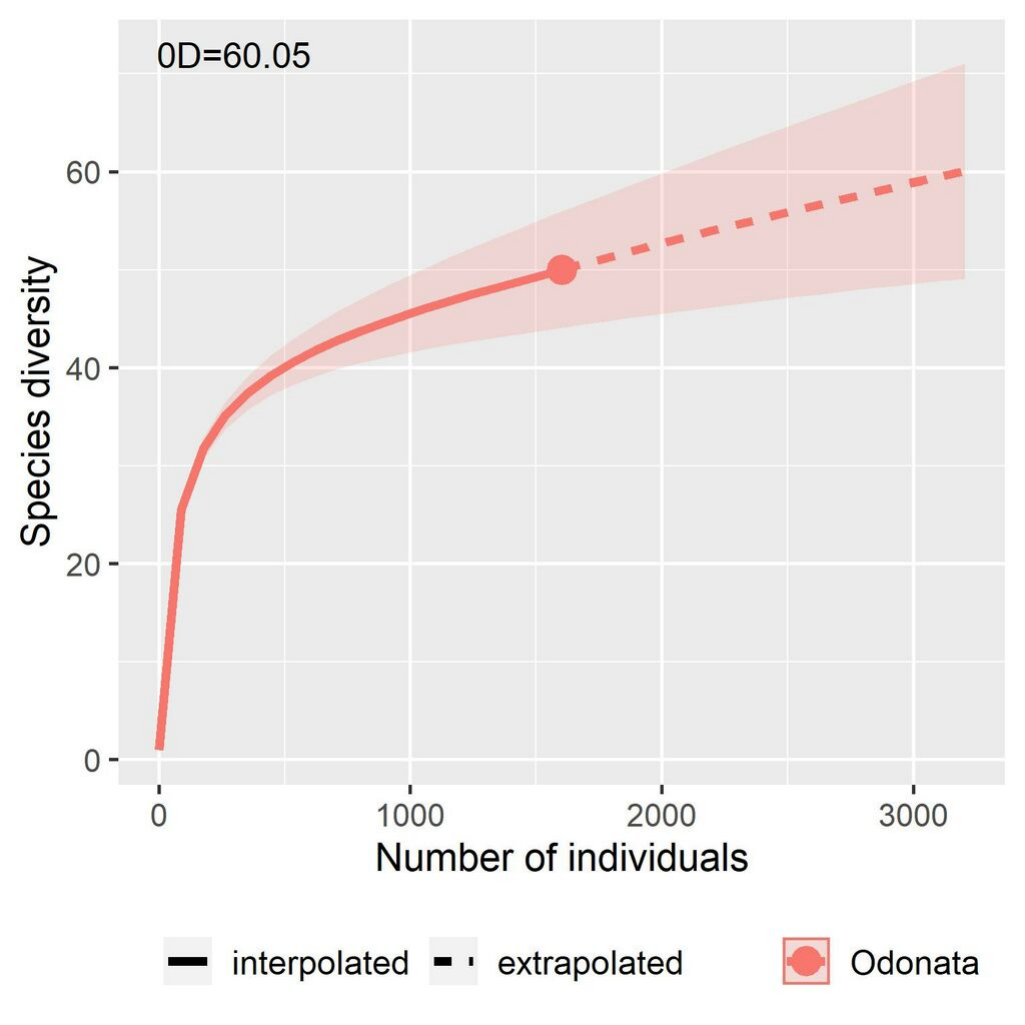
Species accumulation curve (interpolation-extrapolation) for the Dominguillo Odonata assemblage, based on species abundance. ^0^D is the expected number of species according to this curve.

The expected richness for the whole assemblage was between 83.26% (60.05 species) and 64.5% (77.48 species) versus the richness of 50 observed species, calculated through the interpolation-extrapolation method and the Chao1-*bias corrected* estimator, respectively (Fig. 3).

Our estimations indicated that more sampling efforts in Dominguillo are necessary in order to obtain more species, although, based on the collector experience of one of us (EGS), we suggest that the more probable scenario to be expected might be the one calculated through the interpolation-extrapolation method.

**Figure 4.**
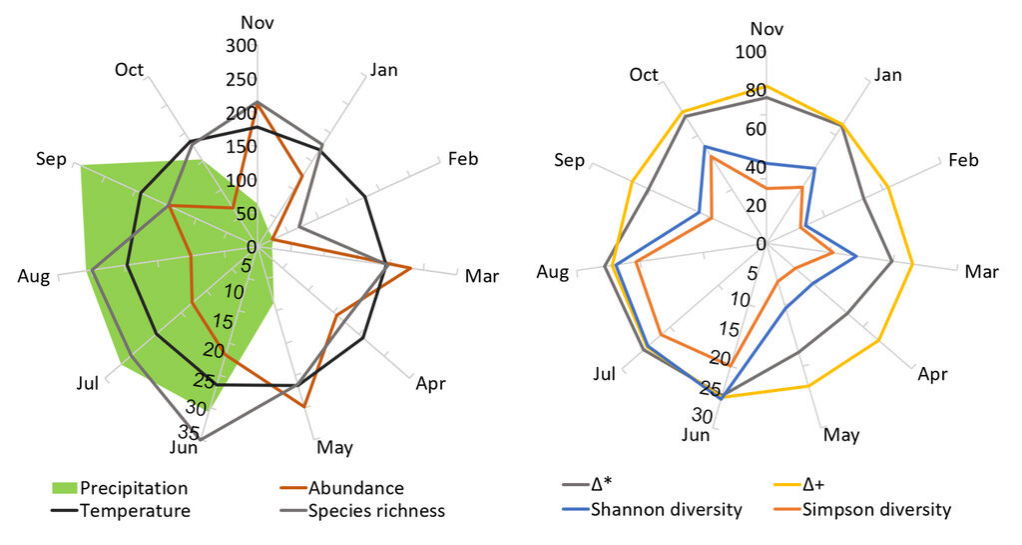
Monthly values for abundance, diversity and taxonomic distinctness (phylogenetic divergence) of the Odonata from Dominguillo and their relation with monthly average precipitation and temperature. Shannon diversity (*r* = 0.721, P = 0.012), Simpson diversity (*r* = 0.750, P = 0.008), taxonomic distinctness (*r* = 0.684, P = 0.020) and average taxonomic distinctness (*r* = 0.639, P = 0.034) were significant and positively correlated with the average monthly precipitation. Scales added in the November axis correspond to precipitation, abundance and phylogenetic diversity variables; scales added in the June axis correspond to temperature, species richness, Shannon and Simpson diversities.

On the other hand, the value for Shannon diversity (^1^D) throughout the year was 21.07 effective species and 13.17 for the Simpson diversity or evenness (^2^D), while the estimated values of effective species for those same metrics were 21.64 and 13.27, respectively (Fig. 4).

#### Phenology

Despite Dominguillo’s ecosystem seasonality, Odonata richness and abundance did not show a pattern of seasonality, as has been observed in other insect groups.

**Figure 5.**
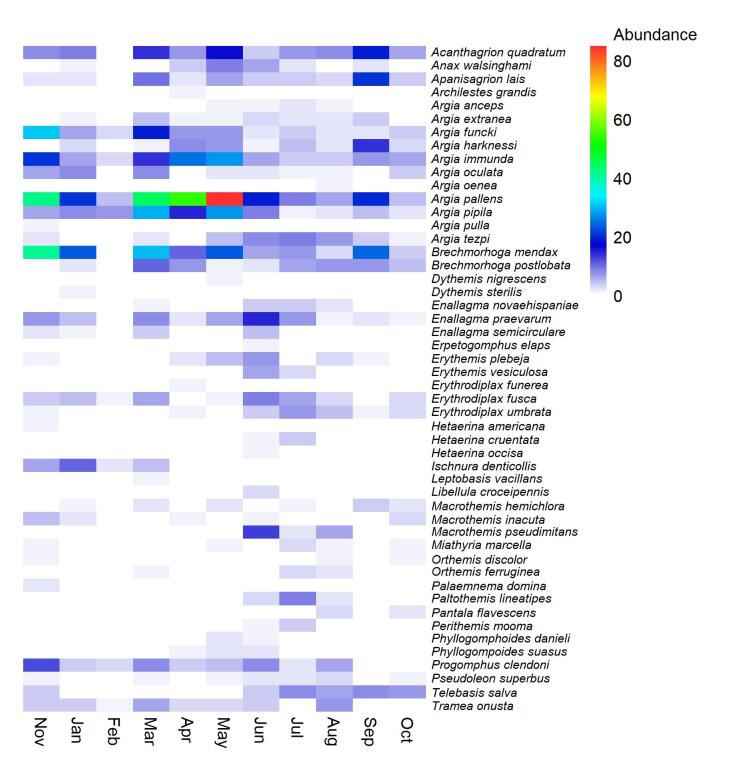
Heatmap displaying the variation in monthly abundance of adult flying Odonata from Dominguillo, Oaxaca.

The highest values of abundance were recorded during the dry season, in March and May, while February was the month with the lowest abundance, followed by October (Fig. 4). It is important to note here that the peaks of abundance in March and May were mainly caused by a few species (Fig. 4). A total of 23 species were recorded in March, but of those, *Argia pulla* (45 individuals), *Hetaerina americana* (30 individuals) and *A. tezpi* (28 individuals) made up 45% of all the individuals registered in that month; 25 species were recorded in May, of which *Argia pulla* (85 individuals), *A. tezpi*, *A. oenea* (28 individuals each) and *H. americana* (23 individuals) made up 66% of the individuals collected that month. In fact, the monthly pattern of high dominance — with just a few species being highly abundant and most species being very scarce — was more evident in the dry season than in the rainy season, as was shown through ^2^D (Figs 4, 5).

The highest richness (^0^D) was recorded early in the rainy season, during June, July and August (34, 29 and 29, respectively), but in September — still during the rainy season — a low species richness was also recorded (Fig. 4). The lowest value was recorded in February, during the dry season, followed by September (Fig. 4). The ^1^D diversity changed every month: the lowest value was recorded in May (6.69 effective species) and the highest from June (25.38) to August (23.79), coinciding with the rainy season (Table 2). On the other hand, diversity ^2^D showed the lowest value in February (5.84) and the highest in July (21.85) (Fig. 4Table 2). Evenness amongst dominant species was also higher during the rainy season than the dry season. Aside from that, the monthly phylogenetic diversity, measured through taxonomic distinctness index (Δ*), was higher than expected (67.59) for all months, except February to May, i.e. the main part of the dry season. In contrast, the average taxonomic distinctness (Δ^+^) for each month was less than that expected in all cases (see Table 2).

Adult activity was very heterogeneous: eigth species flew as adults from 10-11 months, 10 species from 7-9 and 4-6 and 22 species from 1-3 months. Only four species out of the total flew during the entire period (11 months) and opposite to that, 13 species were rare and flew only during one month. The species that flew all year long belong to the *Argia* genus and, with the exception of *Argia funcki* Selys, 1854 and *Argia pipila* Calvert, 1907, which were recorded flying for only 1 and 4 months, respectively, the rest of the species of this genus (4) were recorded flying from 8 to 10 months (see Fig. 5).

Seasonally, 33 species were active in both the rainy and the dry seasons, nine only during the rainy season and eigth only during the dry season. Regarding abundance, 537 individuals were collected in the rainy season and 1066 during the dry season.

#### Relationships between species diversity and phylogenetic divergence with abiotic factors

In our study, the Pearson’s correlation showed that only the Shannon diversity (*r* = 0.721, P = 0.012), the Simpson diversity (*r* = 0.750, P = 0.008), the taxonomic distinctness (*r* = 0.684, P = 0.020) and the average taxonomic distinctness (*r* = 0.639, P = 0.034) were significant and positively correlated with the average monthly precipitation. In other words, as rainfall begins to increase, the diversity and evenness also increase, as well as the phylogenetic divergence (taxonomic relationship between species) of the taxonomic monthly structure within the odonate assemblage. No significant relationship was found between temperature and diversity or phylogenetic divergence.

#### Comparison with other TDF regions

Odonata species richness, recorded for the Dominguillo Region (50 species), was greater than reports from Aguililla, Michoacan (40) (Novelo-Gutiérrez and Gómez-Anaya 2008) and similar to reports from San Javier, Sonora (52) (González-Soriano et al. 2009) and Pinolapa, Michoacan (51) (Novelo-Gutiérrez and Gómez-Anaya 2008). However, they were lower than those from the Sierra de Huautla, Morelos (57) (González-Soriano et al. 2008) and from Chamela, Jalisco (78) (González-Soriano et al. 2004), which is the site with TDF as the dominant vegetation that has the largest number of species recorded to date in Mexico.

The number of species that Dominguillo shares with these localities is variable, but the values are relatively close. Dominguillo shares 29 species (58%) with Aguililla and San Javier each, 32 species (64%) with Huautla and Pinolapa each and 28 species (56%) with Chamela.

## Discussion

Dominguillo is located in the north-eastern part of the State of Oaxaca, in what is known as the Cañada Region. It is part of the Tehuacan-Cuicatlan Biosphere Reserve, an interesting xerophytic area with a large proportion of endemic animal and plant species, but with poor knowledge on its odonate fauna.

Compared to both Huautla and Chamela, the low species richness found in Dominguillo could be the result of sampling around a homogeneous aquatic habitat and in a more restricted area. The reduced number of species of important groups (e.g. Aeshnidae with only one species) also denotes this. In Dominguillo, the Rio de las Vueltas is an exposed rocky stream with a permanent water flow and few zones of lentic backwaters. In contrast, the presence of a dam in the vicinity of sampling sites in Huautla and the occurrence of remnant pools downstream from the dam during the dry season, facilitate the presence of more species associated with lentic habitats, which, in turn, contributes to the increase in the richness of the site (González-Soriano et al. 2008). In relation to Chamela, although samplings were made at intermittent periods, they were carried out during the course of several years and included a wide array of habitats, such as coastal lagoons, swamps and temporal pools, all of which are very favourable for the species of the Coenagrionidae and Libellulidae families (González-Soriano et al. 2004). In addition, Chamela is a well-preserved forest within the Chamela-Cuitzmala Biosphere Preserve. On the other hand, Dominguillo is located in an ecotone between TDF and xerophytic vegetation, which is a much drier area compared to the TDF located in Huautla and Chamela.

A positive relationship between richness, diversity and precipitation has been found in other insect groups at this and other sites (e.g. Noguera et al. 2017). For instance, in several groups of Coleoptera (e.g. Cantharidae, Cerambycidae, Lampyridae, Lycidae and Phengodidae) and Hymenoptera (e.g. Encyrtidae at some sites), the highest number of species coincides with the beginning or the middle of the rainy season (Noguera-Martínez et al. 2012, Rodríguez-Velez et al. 2009, Rodríguez-Velez et al. 2011). In contrast, in San Javier, Sonora and Huautla, Morelos, more species of Encyrtidae were recorded during the dry season (Rodríguez et al. 2010, Rodríguez-Vélez and Wooley 2005). In San Javier, there is a visible peak of odonate adults during the wet season, but richness values in Huautla fluctuate throughout the year (González-Soriano et al. 2008).

In this study, the increase in both diversity and phylogenetic divergence of the odonate assemblage is apparently related to an increase in rainfall. Our results indicate that an increase in rainfall not only influences the distribution of abundance amongst species, but also the identity and/or the type of genera and/or families (i.e. the phylogenetic divergence) that constitute those temporal assemblages. When precipitation increases during the rainy season, the taxonomic distances amongst species also increase. During the wet season, high rainfall probably increases the availability of niches and resources (biotic and abiotic), which brings forth not only a higher number of taxa, but also allows the co-existence of taxa with more diverse ecological requirements. Taxonomic distinctness has been proven to be a highly useful index to evaluate the taxonomic structure of communities and assemblages in other odonates studies (Campbell and Novelo-Gutiérrez 2007).

A close examination of the Dominguillo assemblage (Fig. 4) reveals that the monthly species composition of the community varies throughout the year. Therefore, the increase in phylogenetic divergence of the assemblage observed during the rainy season is due to the recruitment of taxa that were not present early during the dry season. For example, *Archilestes grandis* Rambur, 1842, *Palaemnema domina* Calvert, 1903, *Phyllogomphoides danieli* González & Novelo, 1990, *P. suasus* Selys, 1859 and *Progomphus clendoni* Calvert, 1905 are species that were observed only during the wet season. This sub-group alone results in the addition of four genera, three families and four subfamilies that were not recorded during the dry season. Additionally, *Apanisagrion lais* Brauer in Selys, 1876 (Coenagrionidae) and *Miathyria marcella* Selys in Sagra, 1857 (Libellulidae) are two species belonging to unique genera within the study area that were recorded only during the wet season. All species of the “late spring/summer” group contributed significantly to an increase in the observed phylogenetic diversity during the wet season.

At a local scale, studies in Mexico reveal that the variation in the seasonal composition of the assemblage observed in our study seems to occur also at other localities with TDF (González-Soriano et al. 2009, González-Soriano et al. 2008). For example, several species of Coenagrionidae (especially in the *Argia* genus) and Calopterygidae (*Hetaerina*) are active as adults throughout most of the year in Dominguillo, Huautla and San Javier. On the other hand, species of the Gomphidae family (except perhaps for *Erpetogomphus*, which appears to have a more extended flying period) and the Platystictidae family (at some sites) occurred as adults only during the wet season (see also Campbell and Novelo-Gutiérrez 2007 for another ecosystem). Kishimoto and Itioka (Kishimoto-Yamada and Itioka 2015) mentioned that more long-term studies are necessary in order to understand the causes underlying seasonality in odonates. Here, we add that more inter-site comparative studies will shed some light on understanding the causes for these variations at this and other Mexican tropical dry forests. Unfortunately, climate change predictions for the entire Tehuacán-Cuicatlán Preserve suggest increased aridity, higher temperature and lower rainfall leading to reduced river flow and increased salinity and mineralisation of the drylands streams, leading to a loss of aquatic macroinvertebrates' biodiversity (López-López et al. 2019). According to our results, we suggest that those changes in temperature and precipitation regimes will not only affect odonate abundance and species richness, but also taxonomic structures of the TDF odonate assemblages.

## Conclusions

The odonate assemblage from Santiago Dominguillo, Oaxaca consists of six families, 26 genera and 50 species. However, our data analysis suggests higher species richness in the region and, therefore, it would be interesting to extend the field work for a longer time in order to test this prediction. Dominguillo odonates did not show a significant relationship between abundance and richness with temporal variation in precipitation, which is opposite to that shown by other TDF insect assemblages. Nonetheless, both the monthly species diversity and the monthly phylogenetic diversity did show a significant relationship with temporal variation in precipitation. No significant relationship was found with temperature. In particular, the seasonal abundance pattern was different from those found in other TDF insects and more efforts should be made to explore their causes and associated factors. The high monthly phylogenetic diversity, registered during the rainy season, indicates a high variation in the temporal taxonomic composition within the odonate assemblage — a pattern that had not been recorded before in this order — and it might be related to ecological factors, such as competition amongst species.

Odonate TDF assemblages have been poorly explored around the world, in spite of the threats to their habitats caused by diverse environmental changes and human activities (Campbell et al. 2010, Sánchez-Bayo and Wyckhuys 2019). Climate change is a factor affecting dryland streams in the semi-arid Tehuacan-Cuicatlan Biosphere Reserve, leading to a loss of aquatic macroinvertebrates diversity (López-López et al. 2019). Here, we suggest that both species richness and taxonomic structures of odonate assemblages will be affected by those changes. Therefore, a better understanding of their spatial and temporal patterns would be helpful to evaluate some of the most relevant threatening factors and to design and plan relevant conservation strategies.

## Supplementary Material

B6DBA119-B301-54CB-A819-556A3668E1E2 10.3897/BDJ.9.e60980.suppl1 Supplementary material 1 Specimen Database Data type Excel sheet Brief description This file contains the database of specimens of Odonata from Santiago Dominguillo, Oaxaca, Mexico and from which the information used in the analyses presented was extracted.
Our records are deposited in the database of the Biological Collections at the Institute of Biology, National Autonomous University of Mexico (UNAM) and available through the IBdata Portal developed by that institute at: http://www.ibdata.ib.unam.mx/web/web-content/admin-queryfilter/queryfilter.php The records are also available through the Portal de Datos Abiertos, UNAM at: https://datosabiertos.unam.mx/ File: oo_467310.xlsx https://binary.pensoft.net/file/467310 Enrique González-Soriano and Ubaldo Melo-Samper

2513B0DF-942A-53BC-B0CD-9C9536A839F4 10.3897/BDJ.9.e60980.suppl2 Supplementary material 2 Dictionary of database fields Data type Excel sheet Brief description This file contains the description of the fields used in the Odonata database, following the DarwinCore standard. File: oo_467312.xlsx https://binary.pensoft.net/file/467312 Enrique González-Soriano and Ubaldo Melo-Samper

## Figures and Tables

**Table 1.** Species richness by family from the State of Oaxaca and Santiago Dominguillo; in parentheses, the proportion of Dominguillo species in relation to Oaxaca diversity, based on González-Soriano and Novelo-Gutiérrez 2014 and González-Soriano, unpublished data.

**Families**	**Dominguillo**	**Oaxaca**
Lestidae	1 (14.3)	7
Calopterygidae	3 (37.5)	8
Coenagrionidae	19 (35.2)	54
Platystictidae	1 (20)	5
Aeshnidae	1 (12.5)	8
Gomphidae	4 (21)	19
Libellulidae	21 (39.6)	53

**Table 2.** Monthly diversity of the Odonata of Dominguillo, plus expected values of taxonomic distinctness (^a^).

**Sampling months**	**Abundance**	**Species richness (^0^D)**		**Shannon diversity (^1^D)**		**Simpson diversity (^2^D)**		**Phylogenetic divergence**	
		**Obs**	**Est**	**Obs**	**Est**	**Obs**	**Est**	Δ*	**Δ^+^**
Nov	212	25	31.10	12.38	13.39	8.47	8.78	75.50	81.48
Jan	124	21	25.13	13.87	15.44	10.39	11.25	72.60	73.22
Feb	25	8	9.92	6.69	8.17	5.84	7.32	55.68	69.58
Mar	236	23	29.22	14.09	15.04	10.50	10.95	65.96	76.70
Apr	153	20	25.96	9.59	10.47	5.93	6.12	55.64	77.30
May	246	25	31.10	10.60	11.33	6.22	6.35	59.19	77.48
Jun	168	35	41.36	25.38	28.98	19.96	22.52	82.57	83.65
Jul	124	29	29.74	24.53	27.67	21.85	26.14	84.78	82.44
Aug	100	29	31.55	23.79	28.19	20.66	25.78	85.47	81.40
Sep	145	17	17.66	11.64	12.38	9.46	10.05	67.88	77.22
Oct	68	21	25.11	17.91	21.80	16.06	20.71	78.25	81.17
Total	1601	50	77.48	21.07	21.64	13.17	13.27	67.59^a^	84.63^a^
